# Comparison of Smoking Cessation Outcomes in Smokers With Chronic Obstructive Pulmonary Disease, Coronary Artery Disease, Asthma, and Healthy Smokers: A Prospective Study of 400 Participants

**DOI:** 10.7759/cureus.75095

**Published:** 2024-12-04

**Authors:** Mustafa I Bardakci, Mutlu Sumerkan, Gülhan Ayhan Albayrak, Arzu Özkarafakili, Remzi Gediz, Gulcan Sagir

**Affiliations:** 1 Chest Diseases, Şişli Hamidiye Etfal Training and Research Hospital, University of Health Sciences, Istanbul, TUR; 2 Cardiology, Şişli Hamidiye Etfal Training and Research Hospital, University of Health Sciences, Istanbul, TUR; 3 Pulmonology, Şişli Hamidiye Etfal Training and Research Hospital, University of Health Sciences, Istanbul, TUR

**Keywords:** broncheal asthma, cigarette smoke, copd: chronic obstructive pulmonary disease, coronary artery disease, smoking cessation

## Abstract

Objectives: In this study, we aimed to demonstrate the effect of motivational interviewing with a specific cohort of smokers on smoking cessation. Furthermore, we investigated the influence of medical conditions and individual traits on the efficacy of motivational interviewing for smoking cessation.

Methods: This prospective study was conducted with smokers who presented at the pulmonology and cardiology outpatient clinic. Routine tests, Fagerstöm nicotine dependence test, and hospital-scale anxiety-depression test were performed. Smoking cessation motivational interviews were conducted by pulmonologists. Patients were evaluated for smoking cessation at the end of the first, third, and sixth months.

Results: The study encompassed 100 participants from each of the following groups: asthma, chronic obstructive pulmonary disease, coronary artery disease, and healthy individuals with a smoking habit. The median age of the participants was 52 (44 to 58) years. Among the 400 patients, 177 (44.2%) were female, with a median age of 50 (42-58) years, and 223 (55.8%) were male, with a median age of 53 (46-59) years. According to smoking cessation, 85 (21.3%) patients quit smoking at the end of the first month. It was 55 (13.8%) at the end of the third month and 42 (10.5%) at the end of the sixth month. According to gender, the cessation of smoking in the sixth month was significantly higher in females.

Conclusion: We conducted smoking cessation support interviews with patients and succeeded in smoking cessation in 10.5% of the patients at the end of the sixth month. At the end of the first month of follow-up, smoking cessation success was 20%. Success was higher in the female population than in males.

## Introduction

Tobacco is a botanical species indigenous to South America. Tobacco has been utilized not only to smoke but also for chewing, ingestion, topical application, and ocular administration throughout its history, yet smoking remains the most enduring method [1]. With the acknowledgment of its pleasurable attributes, its utilization has proliferated significantly [1].

The World Health Organization (WHO) defines cigarette addiction as a chronic illness characterized by progression and recurring relapses [2]. This epidemic, spanning centuries, has severe consequences in terms of morbidity and mortality [3]. Annually, more than seven million people worldwide and upwards of 100 thousand people in Turkey die as a result of smoking-related diseases [4]. Considering variables such as the age of smoking initiation, duration of use, daily consumption volume, and quality of the product used, it has been observed that as exposure to the dose increases, smoking-related diseases and mortality increase [5].

The age of starting smoking varies between genders, with females typically beginning smoking at a slightly later age than males. The prevalence of smoking is significantly higher among males than females. The male-to-female ratio is highest in Egypt and lowest in Poland and Uruguay [6]. Individuals in Asia and Africa tend to initiate smoking at a later age compared to those in Europe and Latin America [6]. Turkey is one of the 10 countries with the highest smoking rates in the world [7]. According to 2016 data on current smoking rates in Turkey, approximately two out of every five males, one out of every eight females, and an average of one out of every four individuals in the general population are reported to be smokers [8].

The toxins in cigarette smoke permeate the bloodstream and harm nearly every organ in the body [9,10]. Smoking is linked to the onset of chronic obstructive pulmonary disease (COPD), as well as chronic respiratory diseases, such as asthma and interstitial lung disease [11]. Smoking is critical due to its status as the leading preventable factor associated with cardiovascular diseases [12]. The risk of myocardial infarction is directly correlated with the quantity of smoking. Over half of the deaths attributed to coronary artery diseases (CADs) are sudden in nature, and the cessation of tobacco can lead to a decreased risk of sudden death [10].

Quitting smoking can diminish smoking-related diseases and fatalities, decelerate the rate of disease progression, and augment life expectancy [9]. Smoking cessation therapies are extensively utilized, and smokers now have more access to smoking cessation methods than ever before. Smokers who receive smoking cessation treatment, a combination of behavioral interventions and medication, are five times more likely to succeed in quitting compared to those who attempt to quit independently [13].

Numerous studies report disparate findings regarding the factors influencing the success of smoking cessation. Prominent factors include motivation and determination, sociodemographic attributes, addiction severity, psychological and environmental influences, and comorbidities [3]. Approximately 70-80% of tobacco users desire to quit, with approximately one-third attempting to quit independently each year. However, less than 10% succeed in their cessation endeavors [14]. Two approaches have proven beneficial in the endeavor to quit smoking. These approaches encompass motivational interviewing and pharmacotherapy, incorporating behavioral therapy techniques [15].

In their routine clinical practice, physicians ought to inquire of every patient, irrespective of the reason for the visit, regarding their smoking status, the quantity of smoking, and their contemplation of cessation. Additionally, healthcare professionals should support those wishing to quit and conduct motivational interviews with those not yet ready. Research has demonstrated that the mere act of physicians delicately inquires patients' smoking habits and issuing admonitions prompts contemplation toward cessation, culminating in a success rate of 1-3% in quitting [16].

Motivational interviewing is based on identifying and exploring the underlying issues pertinent to smokers, aiming to effect behavioral change. The dynamic is grounded in a collaborative and amicable partnership, diverging from the traditional consultant-client paradigm [17]. Randomized controlled trials have shown that motivational interviewing for smoking cessation yields superior efficacy compared to dispensing brief advice [18].

The pervasive recurrence of cessation failures and relapses underscores the imperative for smokers to pursue professional assistance.

In our study, we administered motivational interviews for smoking cessation to a specific cohort comprising both patients and healthy smokers. Our objective was to demonstrate the impact of motivational interviewing on smoking cessation. Furthermore, we investigated the influence of medical conditions and individual traits on the efficacy of motivational interviewing for smoking cessation.

## Materials and methods

Participants and study design

This prospective study was conducted with smokers who presented at the pulmonology and cardiology outpatient clinic. The study encompassed 100 participants each from asthma, COPD, and healthy individuals with smoking (HIWS) habit cohorts who were admitted to the pulmonary outpatient clinic, along with 100 individuals diagnosed with CAD who attended the cardiology outpatient clinic during the period spanning from April 20, 2023, to July 20, 2023. Our study enrolled healthy individuals of any gender, aged 18 years or older, who had smoked for at least 15 years. Individuals with deficient medical documentation, immune system disorders, malignancy, a second chronic disease other than COPD/asthma/CAD, and a smoking history of less than 15 years were not included in the study. Routine laboratory tests, electrocardiography (ECG), pulmonary function testing (PFT), chest radiography, Fagerstöm nicotine dependence test (FTND), and hospital-scale anxiety-depression test (HAD-A/HAD-D) were performed on patients. In addition, low-dose chest computed tomography (CT) was administered for lung cancer screening among individuals aged over 50 years of age and with a smoking history exceeding 20 pack years.

FTND serves as a standardized instrument for evaluating the degree of physical nicotine dependence. Uysal et al. adapted the FTND into Turkish, confirming its validity and reliability through a study conducted in 2004 [19]. The assessment comprises six items, yielding a cumulative score from 0 to 10. In this study, an FTND score of 9 or higher defined very high dependence, with categorizations ranging from very low (0-2), low (3-4), moderate (5-6), and high levels of dependence levels.

The HAD scale was devised to detect anxiety and depression among patients grappling with physical diseases [20]. The scale comprises a total of 14 items. Of these, seven items measure symptoms indicative of anxiety, while the remaining seven assess symptoms indicative of depression. Following studies conducted in Turkey, the cut-off score for the anxiety subscale (HAD-A) was set at 10, and for the depression subscale (HAD-D), it was established as 7.

Smoking Cessation Motivational Interview

Interviews were conducted by pulmonologists. The pulmonologists gave a concise lecture covering topics such as nicotine addiction, nicotine withdrawal syndromes, the adverse effects of smoking, the advantages of quitting, strategies to manage weight gain, and techniques to navigate trigger situations. Patients were scheduled for outpatient clinic follow-up appointments at the conclusion of the first month, the third month, and the sixth month. In addition, brief support and behavioral interviews were conducted with the patients via telephone weekly for the first month, followed by sessions every 15 days after that. During the follow-up visit, the patient's smoking cessation status was assessed. Patients who were unable to quit smoking received another brief support session. During each visit, discussions averaging about 10 minutes centered on motivational factors, identification of triggers, management of weight gain, and utilization of medications.

Ethical approval

This study was approved by the hospital institution ethics committee (02.05.2023/2306). Consent forms were obtained from all patients.

Statistical analysis

The statistical analysis utilized the IBM SPSS Statistics software, version 17.0 (IBM Corp., Armonk, USA). The Kolmogorov-Smirnov test was used to assess the normal distribution of the data, while the ANOVA test was employed to evaluate the homogeneity of the data. Medians with 25-75th percentiles were reported as continuous variables, discontinuous variables were presented as frequencies and percentages, and a chi-square test was applied. Mann-Whitney U and Kruskal-Wallis H tests evaluated median comparisons. Statistical significance was determined at a P-value below 0.05.

## Results

Demographic characteristics and laboratory findings

The study included 400 smokers, comprising 100 individuals with asthma, 100 with COPD, 100 healthy smokers, and 100 patients with CAD, without gender-based differentiation. The median age of the patient cohort was 52 (44-58) years. Among the 400 patients, 177 (44.2%) were female, with a median age of 50 (42-58) years, and 223 (55.8%) were male, with a median age of 53 (46-59) years. The median body mass index was 29.75 (26.51-31.23) kg/m^2^.

The median age of starting smoking was 18 (15-20) years, and the smoking pack-year history was 33 (25-40). All demographic data, smoking history, laboratory findings, FTND, HAD-A, and HAD-D for all study populations and the patients in the four subgroups are depicted in Tables 1-2.

**Table 1:** Comparison of baseline demographic, clinical characteristics, smoking habits, and laboratory results among asthma, COPD, HIWS, and CAD groups. Data are presented as median (25-75th percentiles) or n (%) unless stated otherwise. P-value compares values of all four groups (asthma, COPD, HIWS, and CAD). COPD, chronic obstructive pulmonary disease; CAD, coronary artery disease; HIWS, healthy individuals with smoking; ALT, alanine transaminase; AS, aspartate transaminase; BMI, body mass index; CRP, C-reactive protein; MLR, monocyte-to-lymphocyte ratio; NLR, neutrophil-to-lymphocyte ratio; PLR, platelet-to-lymphocyte ratio

Parameters	All study population	Asthma	COPD	HIWS	CAD	Chi-square value	P-value
Subjects, n (%)	400 (100)	100 (25)	100 (25)	100 (25)	100 (25)		
Demographics							
Gender, female n (%)	177 (44.3)	61 (61.0)	33 (33.0)	46 (46.0)	37 (37.0)	18.758	<0.0001
Age (years)	52.00 (44.00-58.00)	46.00 (40.25-53.75)	54.00 (49.00-59.00)	47.50 (42.00-54.00)	58.00 (51.00-64.00)	75.758	<0.0001
BMI (kg/m²)	29.75 (26.51-31.23)	28.87 (25.77-33.16)	27.71 (24.99-29.35)	28.10 (25.00-30.46)	30.76 (30.76-31.23)	64.804	<0.0001
Tobacco smoking habits							
Age of starting smoking (years)	18.00 (15.00-20.00)	18.00 (15.00-20.00)	16.00 (14.00-19.00)	15.25 (13.10-17.00)	17.00 (16.00-19.00)	38.935	<0.0001
Smoking pack-year history	33.00 (25.00-40.00)	25.50 (21.00-30.00)	38.50 (32.25-45.00)	28.50 (23.00-37.00)	37.50 (32.00-42.00)	97.867	<0.0001
Laboratory testing							
Creatinine (mg/dl)	0.89 (0.71-0.98)	0.72 (0.63-0.84)	0.82 (0.70-0.96)	0.89 (0.75-1.06)	0.96 (0.56-1.07)	100.744	<0.0001
AST (U/L)	24.00 (18.00-31.00)	19.00 (15.25-22.75)	18.50 (15.00-25.75)	26.50 (19.00-32.00)	29.68 (11.00-31.00)	119.539	<0.0001
ALT (U/L)	26.00 (18.00-27.00)	19.00 (14.00-26.00)	19.00 (15.00-27.00)	26.50 (22.00-31.75)	24.66 (7.00-26.00)	48.851	<0.0001
Leukocytes (10^9^/mL)	7.58 (7.26-9.06)	8.28 (7.28-9.96)	8.53 (7.12-9.79)	7.60 (6.74-8.70)	7.37 (4.83-10.36)	43.877	<0.0001
Hemoglobin (g/dL)	14.10 (13.10-14.50)	13.55 (12.52-14.80)	14.45 (13.10-15.70)	13.75 (12.90-14.20)	13.98 (10.20-15.90)	21.322	<0.0001
Hemotocrit	39.80 (38.90-43.40)	41.25 (37.85-43.90)	43.10 (39.75-46.05)	40.15 (37.52-43.12)	39.73 (32.80-46.10)	31.453	<0.0001
Thrombocytes (10^9^/mL)	258.00 (217.25-289.75)	270.00 (220.75-321.00)	265.50 (223.25-325.75)	214.00 (179.75-258.00)	258.64 (166.00-415.00)	42.598	<0.0001
NLR	2.74 (1.95-3.24)	2.10 (1.69-2.69)	2.18 (1.56-2.97)	2.82 (2.32-3.26)	3.12 (1.24-3.24)	87.965	<0.0001
PLR	159.47 (106.19-168.20)	106.97 (90.82-146.68)	110.73 (90.58-140.14)	185.05 (160.60-214.60)	162.59 (77.80-168.20)	147.862	<0.0001
MLR	0.26 (0.19-0.27)	0.22 (0.17-0.28)	0.24 (0.18-0.31)	0.24 (0.19-0.28)	0.25 (0.11-0.33)	9.070	<0.0001
CRP (mg/L)	4.15 (3.50-6.59)	4.35 (1.87-9.57)	5.75 (3.13-8.52)	4.82 (3.71-6.48)	4.19 (0.92-9.17)	16.261	0.001

**Table 2:** Comparison of pulmonary function test findings, FTND, HAD-A, and HAD-D among asthma, COPD, HIWS, and CAD groups. Data are presented as median (25-75th percentiles) or n (%) unless stated otherwise. P-value compares values of all four groups (asthma, COPD, HIWS, and CAD). COPD, chronic obstructive pulmonary disease; CAD; coronary artery disease; HIWS, healthy individuals with smoking; FEV1, forced expiratory volume in one second; FVC, forced vital capacity; FTND, Fagerstrom test for nicotine dependence; HAD-A, hospital anxiety scale; HAD-D, hospital depression scale

Parameters	All study population	Asthma	COPD	HIWS	CAD	Chi-square value	P-value
Subjects, n (%)	400 (100)	100 (25)	100 (25)	100 (25)	100 (25)		
Pulmonary function parameters							
FEV1 %	74.00 (69.00-85.00)	74.50 (64.00-83.00)	60.50 (49.25-71.00)	92.00 (86.00-100.75)	74.00 (69.00-74.00)	223.172	<0.0001
FEV1 (L)	2.12 (1.57-2.56)	2.13 (1.66-2.65)	1.70 (1.35-2.14)	2.93 (2.32-3.41)	2.12 (1.47-2.12)	127.848	<0.0001
FVC (L)	2.76 (2.05-3.20)	2.66 (2.09-3.32)	2.25 (1.85-2.71)	3.42 (2.69-4.11)	2.91 (1.79-2.91)	80.144	<0.0001
FVC %	80.00 (71.00-87.00)	79.00 (67.00-87.00)	65.00 (54.00-74.00)	92.00 (100.75-85.00)	80.00 (71.00-80.00)	193.021	<0.0001
FEV1/FVC ratio (%)	80.00 (74.00-85.00)	83.00 (76.00-86.00)	76.00 (66.00-81.75)	84.50 (87.75-80.00)	74.00 (74.00-82.00)	84.378	<0.0001
FTND						50.467	<0.0001
Very low dependence (0-2), n (%)	6 (1.5)	4 (4.0)	0 (0.0)	2 (2.0)	0 (0.0)		
Low dependence (3-4), n (%)	21 (6.8)	11 (11.0)	1 (1.0)	13 (13.0)	2 (2.0)		
Medium dependence (5), n (%)	51 (12.8)	17 (17.0)	8 (8.0)	20 (20.0)	6 (6.0)		
High dependence (6-7), n (%)	194 (48.5)	45 (45.0)	51 (51.0)	48 (48.0)	50 (50.0)		
Very high dependence (8-10), n (%)	122 (30.5)	23 (23.0)	40 (40.0)	17 (17.0)	42 (42.0)		
HAD-A	8.00 (7.00-9.00)	8.00 (7.00-9.00)	9.00 (8.00-9.00)	8.00 (6.00-9.00)	9.00 (8.00-9.00)	16.732	0.001
HAD-D	6.00 (5.25-7.00)	7.00 (6.00-8.00)	6.00 (5.00-6.75)	6.00 (6.00-7.00)	6.00 (6.00-7.00)	21.143	<0.0001

Smoking cessation data

According to smoking cessation, 85 (21.3%) patients quit smoking at the end of the first month. It was 55 (13.8%) at the end of the third month and 42 (10.5%) at the end of the sixth month. Within all subgroups, the smoking cessation ratios were higher at the end of the first month than in the third and sixth months, with the asthma subgroup exhibiting a particularly notable increase. Although, by the end of the sixth month, smoking cessation rates declined across all subgroups, the most notable smoking cessation rate was observed among the asthma subgroup, with a ratio of 3.5%, but it was statistically insignificant (Table 3).

**Table 3:** Comparison of smoking cessation among asthma, COPD, HIWS, and CAD groups. Data are presented as n (%). P-value compares values of all four groups (asthma, COPD, HIWS, and CAD). COPD, chronic obstructive pulmonary disease; CAD, coronary artery disease; HIWS, healthy individuals with smoking

Parameters	All study population	Asthma	COPD	HIWS	CAD	Chi-square value	P-value
Subjects, n (%)	400 (100)	100 (25)	100 (25)	100 (25)	100 (25)		
Smoking cessation, n (%)							
First month	85 (21.3)	28 (7.0)	23 (5.8)	16 (4.0)	18 (4.5)	5.184	0.159
Third month	55 (13.8)	17 (4.2)	16 (4.0)	11 (2.8)	11 (2.8)	2.593	0.459
Sixth month	42 (10.5)	14 (3.5)	9 (2.2)	10 (2.5)	9 (2.2)	1.809	0.613

At the end of the sixth month, all variables within the study population, encompassing age, age of smoking initiation, smoking quantity, FTND, HAD-A, and HAD-D, were examined concerning smoking cessation. Upon analyzing these variables, no statistically significant difference was identified between individuals who had ceased smoking and current smokers. Conversely, females (26, 61.9%) demonstrated superior outcomes in smoking cessation compared to males (16, 38.1%). In addition, the majority of individuals who quit smoking had a high dependence on nicotine, but it was statistically insignificant (Table 4).

**Table 4:** Comparison of smoking cessation in subjects after the sixth month with demographics and smoking habits. Data are presented as median (25-75th percentiles) or n (%) unless stated otherwise. FTND, Fagerstrom test for nicotine dependence; HAD-A, hospital anxiety scale; HAD-D, hospital depression scale; ¥, Chi-square value; *, Z-value

Parameters	Current smoker	Quit smoking	Chi-square/Z-value	P-value
Subjects, n (%)	358 (89.5)	42 (10.5)		
Demographics				
Gender, female n (%)	151 (42.2)	26 (61.9)	5.929^¥^	0.015
Age (years)	52.00 (44.00-59.00)	49.00 (45.00-55.25)	-1.496*	0.135
Age of starting smoking (years)	18.00 (16.00-20.00)	17.50 (14.75-20.00)	-0.135*	0.892
Smoking pack-year history	34.00 (25.00-41.00)	29.00 (25.00-35.00)	-2.282*	0.022
FTND			4.3^¥^	0.367
Very low dependence (0-2), n (%)	5 (1.4)	1 (2.4)		
Low dependence (3-4), n (%)	25 (7.0)	2 (4.8)		
Medium dependence (5), n (%)	46 (12.8)	5 (11.9)		
High dependence (6-7), n (%)	168 (46.9)	26 (61.9)		
Very high dependence (8-10), n (%)	114 (31.8)	8 (19.0)		
HAD-A	8.00 (7.00-9.00)	8.00 (7.00-9.00)	-1.679*	0.093
HAD-D	6.00 (5.00-7.00)	6.50 (6.00-8.00)	1.803*	0.071

A linear correlation was observed between the first month of smoking cessation (p=0.007) and smoking pack-year history (p=0.025) (Table 5). Furthermore, a significant correlation was found between the third month of smoking cessation and HAD-D (p=0.027). A difference was found between the third month of smoking cessation in median values of smoking pack-year history, but it was not statistically significant (p=0.053) (Table 6). Furthermore, a relationship was found between the sixth month of smoking cessation and gender (p=0.015) and smoking pack-year history (p=0.022) (Table 4). Females exhibited a higher propensity to cease smoking. As the smoking rate decreased, the smoking cessation rate increased. However, according to smoking cessation, no difference was found between subgroups in the first month (Table 3).

**Table 5:** Comparison of smoking cessation in subjects after the first month with demographics and smoking habits. Data are presented as median (25-75th percentiles) or n (%) unless stated otherwise. HAD-D, hospital depression scale

Parameters	Current smoker	Quit smoking	Z-value	P-value
Subjects, n (%)	315 (78.8)	85 (21.3)		
Smoking pack-year history	34.00 (25.00-41.00)	31.00 (25.00-36.00)	-2.242	0.025
HAD-D	6.00 (5.00-7.00)	7.00 (6.00-8.00)	-2.677	0.007

**Table 6:** Comparison of smoking cessation in subjects after the third month with demographics and smoking habits. Data are presented as median (25-75th percentiles) or n (%) unless stated otherwise. HAD-D, hospital depression scale

Parameters	Current smoker	Quit smoking	Z-value	P value
Subjects, n (%)	345 (86.3)	55 (13.8)		
Smoking pack-year history	34.00 (25.00-41.00)	31.00 (25.00-35.00)	-1.938	0.053
HAD-D	6.00 (5.00-7.00)	7.00 (6.00-8.00)	-2.207	0.027

## Discussion

The WHO recognizes smoking as one of the leading preventable health hazards [2]. Numerous methodologies have been devised to aid patients in the endeavor of smoking cessation. In our investigation, we conducted interviews focused on smoking cessation. More than 20% of the smokers subjected to interviews successfully quit smoking by the conclusion of the initial month. It was noted that 50% of these individuals restarted smoking during the subsequent follow-up. The smoking cessation rate among the interviewed cohort was 10.5% by the conclusion of the sixth month. According to gender, the cessation of smoking in the sixth month was significantly higher in females.

The global estimate of tobacco smokers is approximately 1.3 to 1.5 billion individuals. Turkey is among the 10 nations where one-third of global smokers reside [7]. We investigated the effectiveness of smoking cessation interviews in Turkey, which has a high prevalence of smokers. In this context, such endeavors can contribute to reducing smoking prevalence. The adequacy of our patient cohort led us to anticipate greater clarity in the information obtained. Additionally, we devised strategies to elucidate differences by incorporating various patient cohorts into the study design.

Numerous studies document disparate findings regarding the determinants influencing the efficacy of smoking cessation efforts [3]. Approximately 70-80% of tobacco users desire to quit, with approximately one-third endeavoring to do so independently each year. Nevertheless, the success rate is less than 10% [14]. In a study conducted in Taiwan, it was demonstrated that augmenting one's comprehension regarding the deleterious ramifications of smoking within the context of transtheoretical model (TTM)-based smoking cessation interventions markedly enhanced the endeavor to cease smoking [21]. In an alternative study, the efficacy of the health belief model and the TTM model in smoking cessation was evaluated, determining that both methodologies were effective, albeit with the TTM model demonstrating superior effectiveness [22]. In the study by Sharifirad et al., the smoking cessation rate after a six-month follow-up period was revealed to be 3.3% in the control group and 46.0% in the intervention group. Notably, all components of the TTM model yielded significant outcomes [23]. A meta-analysis study concluded that motivational interviews are effective in quitting smoking [24]. Numerous additional studies have corroborated the effectiveness of motivational interviews as a valuable method for facilitating smoking cessation efforts [25-27]. In our research, following the smoking cessation interview and regular follow-up sessions, the success rate for smoking cessation was 21% at the end of the first month. At the end of the sixth month, our smoking cessation rate declined to 10.5%. The main reason for this decline could potentially be attributed to a dilution in patient follow-up after the initial month.

In our study, during the initial evaluation at the outpatient clinic, comprehensive inquiries were made regarding the demographic profiles of individuals and smoking histories. The patients were also assessed using respiratory function tests, FNBT, and the HAD-A/HAD-D scales. The data were compared with those at the end of the sixth month. Studies have demonstrated that certain sociodemographic attributes, including male gender, older age, higher education attainment, elevated socioeconomic status, employment status, and having a stable partnership (such as being married or cohabiting), positively influence the success of quitting smoking [3,9]. Conversely, there are also publications asserting that no discernible gender disparity exists in smoking cessation outcomes [28]. Studies have indicated that varenicline treatment exhibits greater efficacy in facilitating smoking cessation among females compared to males. Conversely, nicotine replacement therapy and bupropion treatment demonstrated a lower success rate in smoking cessation among females relative to males [29]. Enhancing smoking cessation rates among women could be achievable through the implementation of gender-specific studies [30]. One study from China observed variations in smoking behaviors based on gender and rural-urban distinctions [31]. Monsó et al. demonstrated that age, gender, and housing conditions exert a notable influence on smoking cessation outcomes among European participants engaged in smoking cessation programs [32]. Our study gave results that were diverged from those reported in previous studies. In our study, after the sixth month, females exhibited higher smoking cessation rates compared to males. Females had a twofold propensity to quit smoking compared to males. Within the disease categories, the most notable smoking cessation rate was observed within the asthma cohort. This trend could be attributed to the elevated presence of females in the asthma cohort.

Several studies indicate that the duration of tobacco use serves as a pivotal factor in the efficacy of cessation efforts. In the study conducted by Górecka et al., smoking duration emerged as a significant variable influencing the likelihood of successful cessation [33]. In a study conducted in Turkey, it was discovered that individuals who commenced smoking prior to the age of 15 exhibited a markedly reduced likelihood of smoking cessation compared to those who initiated tobacco use later [34]. Likewise, in our study, a correlation was observed between the quantity of tobacco consumed and the cessation status of smoking. However, no significant association was detected between the age of smoking initiation. Numerous studies in the literature assess the correlation between smoking cessation status and FNBT, HAD-A, and HAD-D scores. While some studies have reported a significant relationship, others have not. In our study, a statistically significant relationship was identified between smoking cessation status and HAD-D (in the first and third months of control), but not with FNBT and HAD-A scores.

Study outcomes vary regarding the impact of chronic disease presence on the efficacy of smoking cessation. The study by Salepçi et al. found that the presence of comorbidities among smokers did not influence their success in quitting [35]. Conversely, Uzaslan et al. found that the participants' health problems positively influenced the success of smoking cessation endeavors [36]. Komiyama et al. reported that the success rate of smoking cessation among COPD patients, psychiatric patients, females, and minors was low [13]. In addition, Perret et al. elucidated the relationship between asthma and smoking in their review [37]. Furthermore, Gratziou et al. demonstrated the importance of an intensive smoking cessation program with regular and prolonged follow-ups for smokers with asthma or COPD. Regular and frequent follow-up visits, particularly within the initial three months, were positively correlated with cessation success [38]. Ludvig et al., in their review, compared smoking cessation success rates among patients with CVD and noted that the success rate during treatment reached up to 25%. However, they also noted that the relapse rates were notably high [39]. A meta-analysis by Taylor et al. highlighted that the success rate could increase to 45% with diligent follow-up and cardiac rehabilitation for patients following acute coronary syndrome [40]. In our study, no difference was observed among disease subgroups (asthma, COPD, HIWS, CVD) regarding smoking cessation. The highest rates of smoking cessation were observed during the initial month of intensive follow-up. Consistent with numerous studies, the smoking cessation rates at six months were 10%.

Limitations

We did not experience any significant limitations in our study. The only limitation was the reluctance of patients to receive regular calls and regular check-ups. Although they liked to have a health check-up and be followed up for health, they had a hard time adapting.

## Conclusions

When we evaluated our study together with other studies, we thought that motivational interviewing was effective in smoking cessation treatment. We interviewed our patients for smoking cessation support, and at the end of the first month, we achieved success in about a quarter of them. At the end of the sixth month, this success rate decreased to around 10%. Considering that patient interviews were made more frequently in the first months, it is suggested that these rates may not change if patient follow-ups are made frequently in other months. Smoking cessation success was higher in the female population than in males. In addition, the smoking cessation rate was higher in the first months in groups with respiratory disease. Throughout the entire study, the asthmatic patient group showed a higher smoking cessation success than the other groups. It is evident that supporting motivational interviewing with psychosocial and quality-of-life support tests would increase smoking cessation.
